# A Pediatric Case of Nonprogressive Idiopathic Central Vasculitis: A Spectrum of Clinical Presentations

**DOI:** 10.7759/cureus.71380

**Published:** 2024-10-13

**Authors:** Takuro Nozaki, Kouki Tomari, Tsuyoshi Matsuoka, Hirohisa Taketomi

**Affiliations:** 1 Department of Pediatrics, Okinawa Prefectural Miyako Hospital, Miyako, JPN; 2 Department of General Pediatrics, Okinawa Prefectural Nanbu Medical Center and Children's Medical Center, Haebaru-cho, JPN; 3 Department of Child Neurology and Child Psychiatry, Okinawa Prefectural Nanbu Medical Center and Children's Medical Center, Haebaru-cho, JPN

**Keywords:** idiopathic central vasculitis, immunosuppressive therapy, nonprogressive, primary central nervous system vasculitis, reversible cerebral vasoconstriction syndrome (rcvs)

## Abstract

In children, the causes of cerebral infarction are varied, and accurate diagnosis and treatment are imperative. An early school-age boy was brought to our hospital due to seizures and impaired consciousness. He was diagnosed with cerebral infarction due to primary central nervous system vasculitis (PCNSV) based on increased inflammatory response and circumferential vessel wall thickening in his right middle cerebral artery. As the neurological abnormalities had already improved at the time of diagnosis, he was administered conservative treatment without worsening. This treatment is an option for patients with clinically nonprogressive PCNSV. Some cases of clinically nonprogressive PCNSV and reversible vasoconstriction syndrome can have similar clinical presentations.

## Introduction

The main challenges in treating primary central nervous system vasculitis (PCNSV) are the heterogeneity of the disease and the lack of a standardized treatment protocol. PCNSV is difficult to diagnose because of its nonspecific clinical symptoms and the need for invasive procedures such as brain biopsy for definitive diagnosis. Even in cases confirmed by biopsy, imaging and angiography often yield normal results, complicating the diagnostic process [1]. Although glucocorticoids and cyclophosphamide are commonly used and are effective in many cases, a significant proportion of patients relapse or do not respond adequately to these treatments. For example, in one study, 27% of patients relapsed, and it was reported that relapse was more common with prednisone monotherapy [2]. Cyclophosphamide has also been shown to be effective in inducing remission and reducing relapse when used in combination with glucocorticoids, but it carries a significant risk of toxicity [2-4]. The optimal duration of immunosuppressive therapy and combination therapy remains unclear. Therefore, further research and case accumulation are needed to optimize treatment plans and management strategies tailored to individual patients.

## Case presentation

An early school-age boy who had been healthy suddenly fell, complained of a headache, and was later brought to the hospital for emergency treatment due to impaired consciousness. He had no history of a preceding or prolonged headache other than this headache, and no history of trauma or medication. A significant family history was denied. A physical examination conducted on admission revealed no fever and no obvious trauma scars. The patient was asleep and opened his eyes to painful stimulation. There were no other apparent neurological abnormalities. A head computed tomography (CT) scan revealed no intracranial lesions, such as traumatic brain contusion or subarachnoid hemorrhage. The patient was admitted for observation of suspected prolonged post-ictal unconsciousness. Within six hours after admission, the patient awoke, became alert, and was able to walk independently. Considered the first epileptic seizure, cerebrospinal fluid examination was not performed.

**Figure 1:**
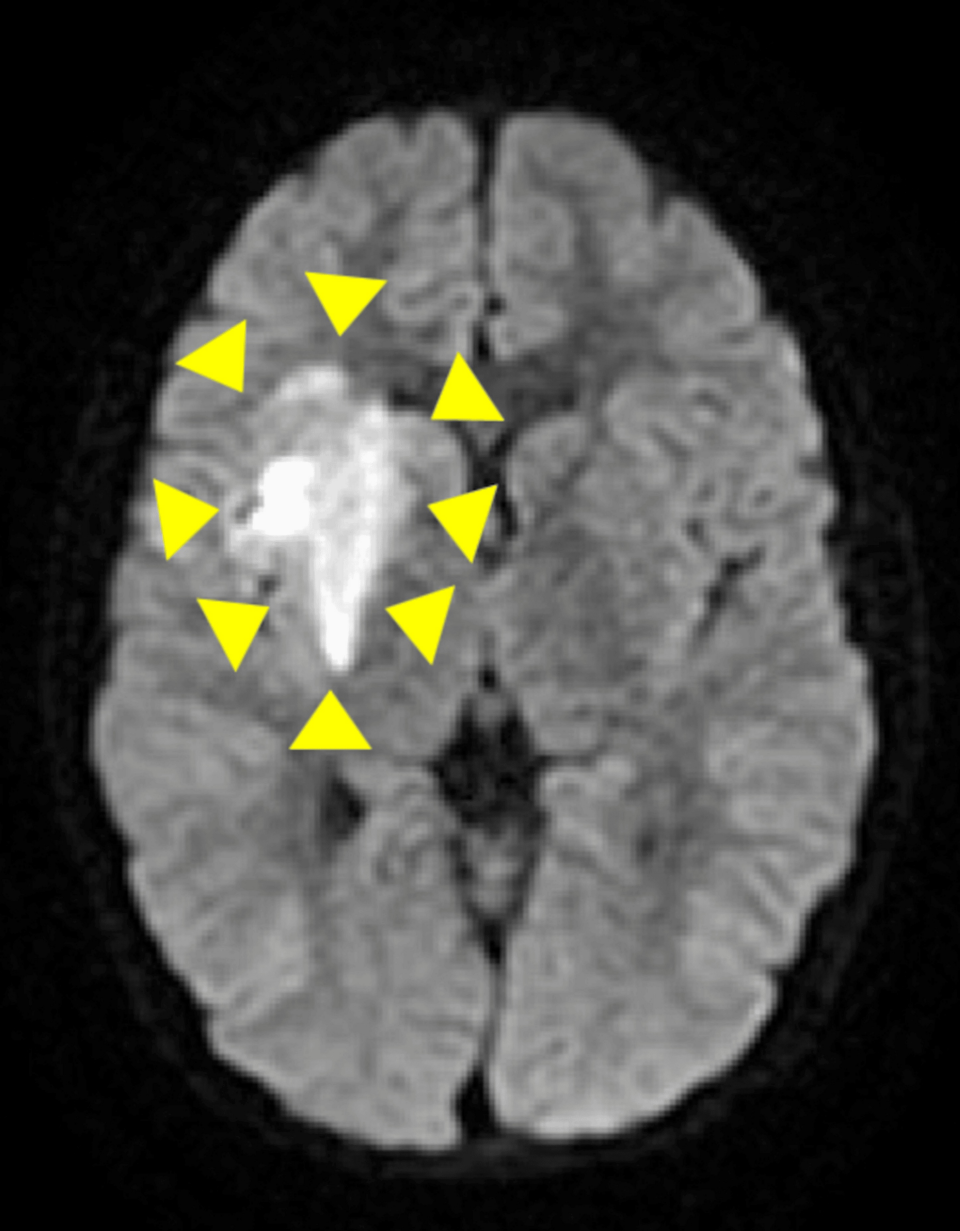
Diffusion-weighted MRI image of the head on day 2 of admission. There is an area of high signal, indicative of cerebral infarction in the right middle cerebral artery (area surrounded by yellow arrowheads).

**Figure 2:**
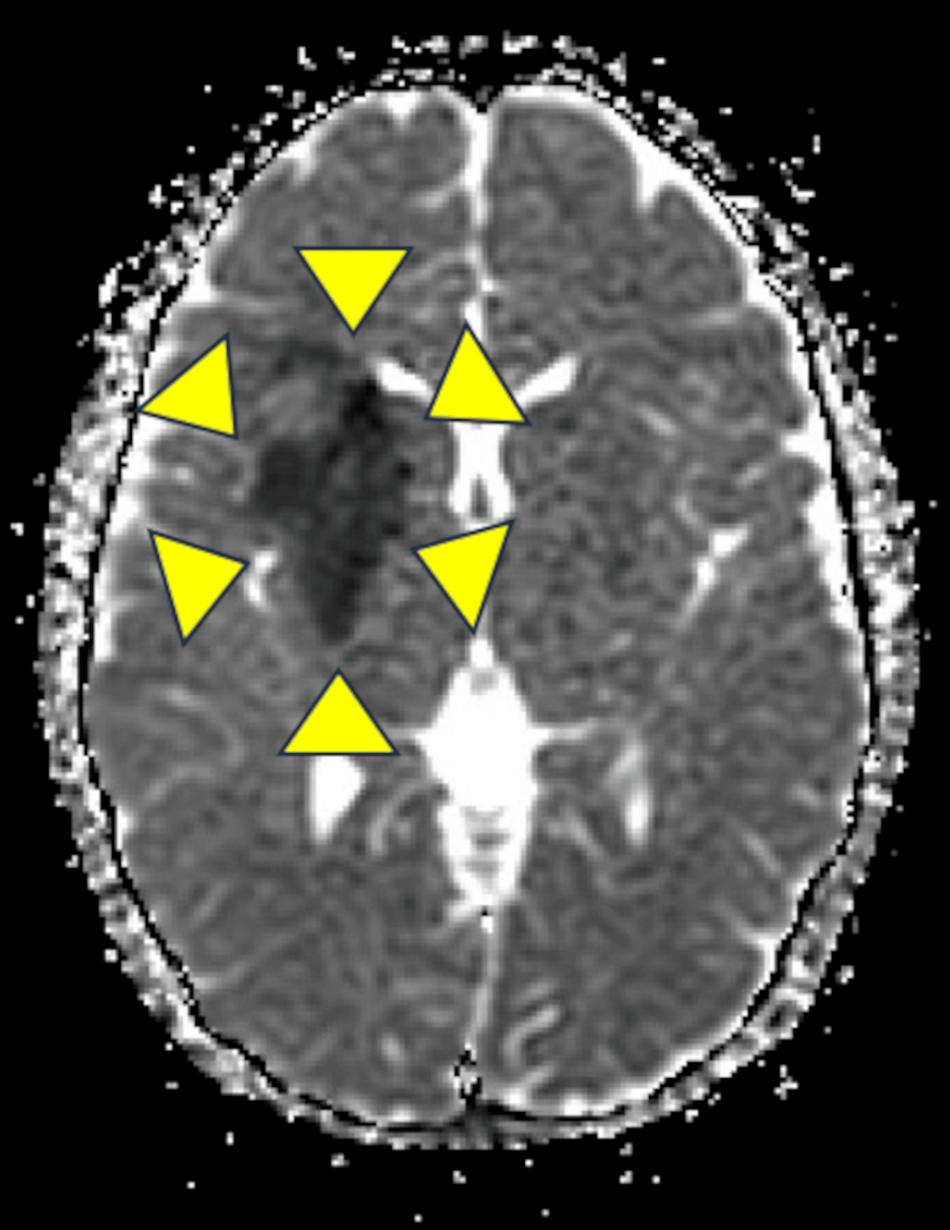
Apparent diffusion coefficient (ADC) MRI image of the head on day 2 of admission. There is an area of low signal, indicative of cerebral infarction in the right middle cerebral artery (area surrounded by yellow arrowheads).

**Figure 3:**
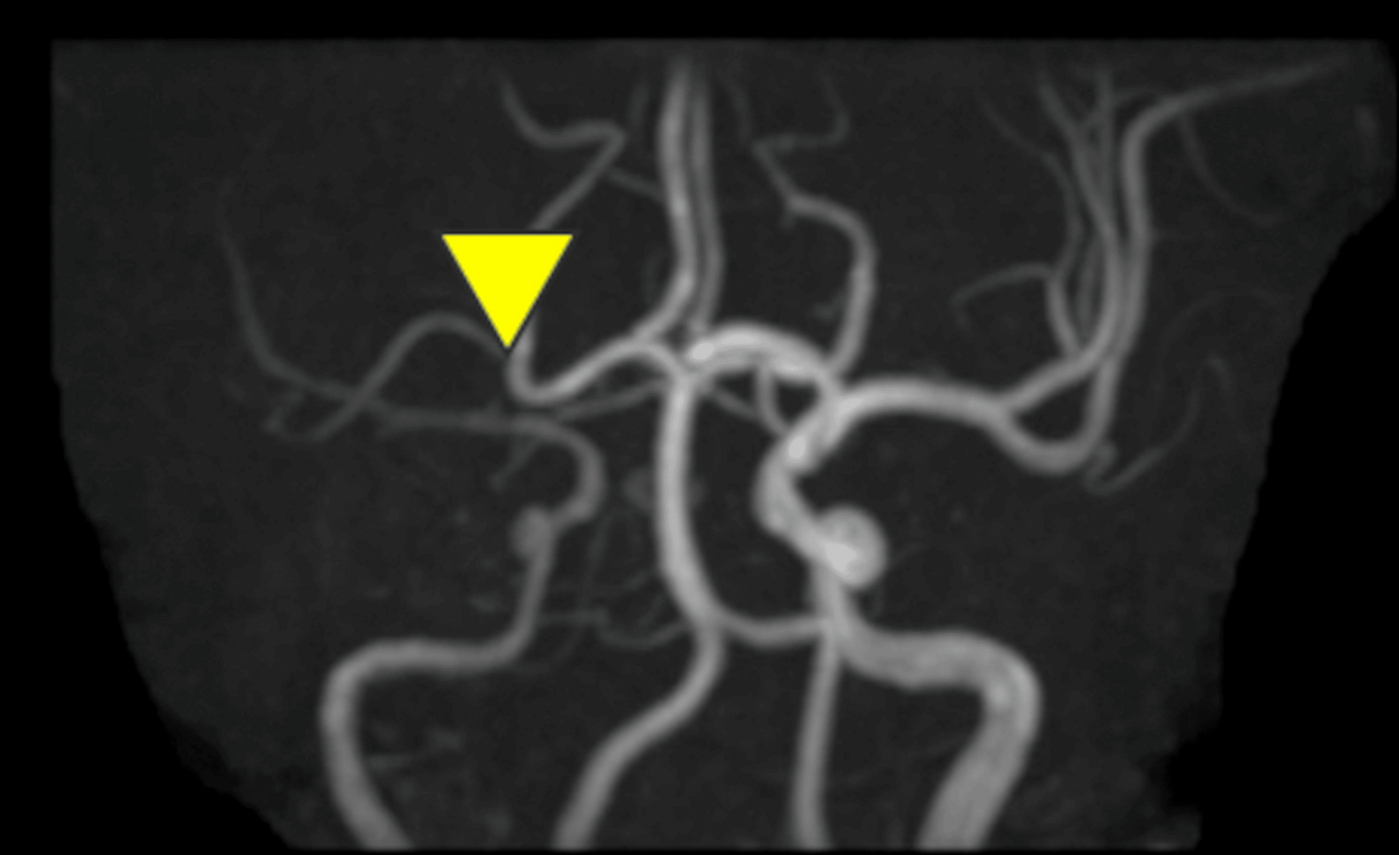
Magnetic resonance angiography of the head on day 2 of admission shows occlusion of the right middle cerebral artery.

On the morning of the second day of admission, incomplete paralysis of the left side of the patient’s body was observed. Magnetic resonance imaging (MRI) of the head was conducted. Diffusion-weighted MRI of the head showed a high signal and apparent diffusion coefficient (ADC) showed a low signal (Figures 1-2), and magnetic resonance angiography (MRA) demonstrated disrupted blood flow in the right middle cerebral artery (Figure 3).

**Figure 4:**
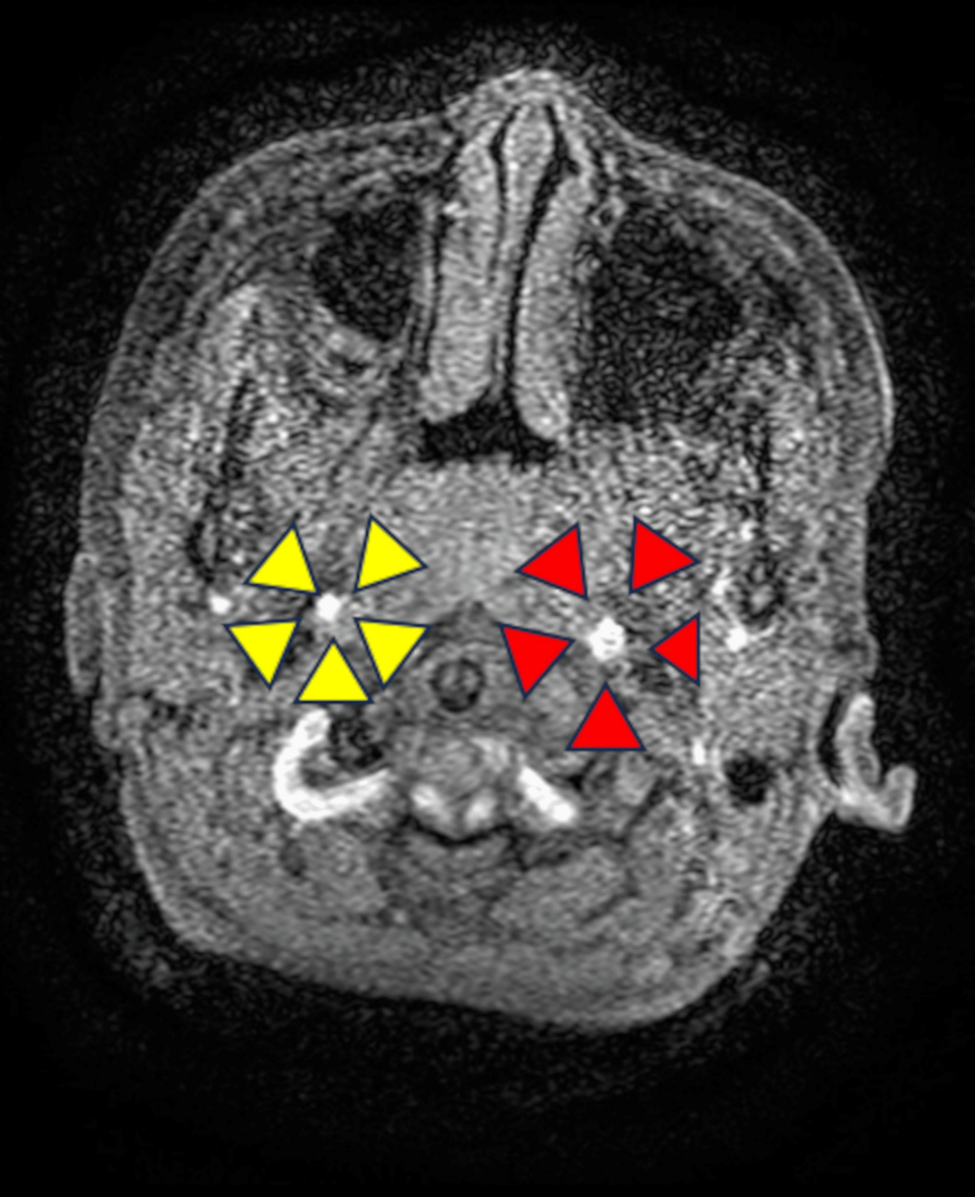
Magnetic resonance angiography of the head on day 12 of admission. On day 12 of the patient's hospitalization, a head MRI angiogram showed a difference in the diameter of the middle cerebral artery on the left and right sides. The diameter on the right (yellow arrowheads) was smaller than that on the left (red arrowheads), indicating a narrowing of the lumen of the artery.

**Figure 5:**
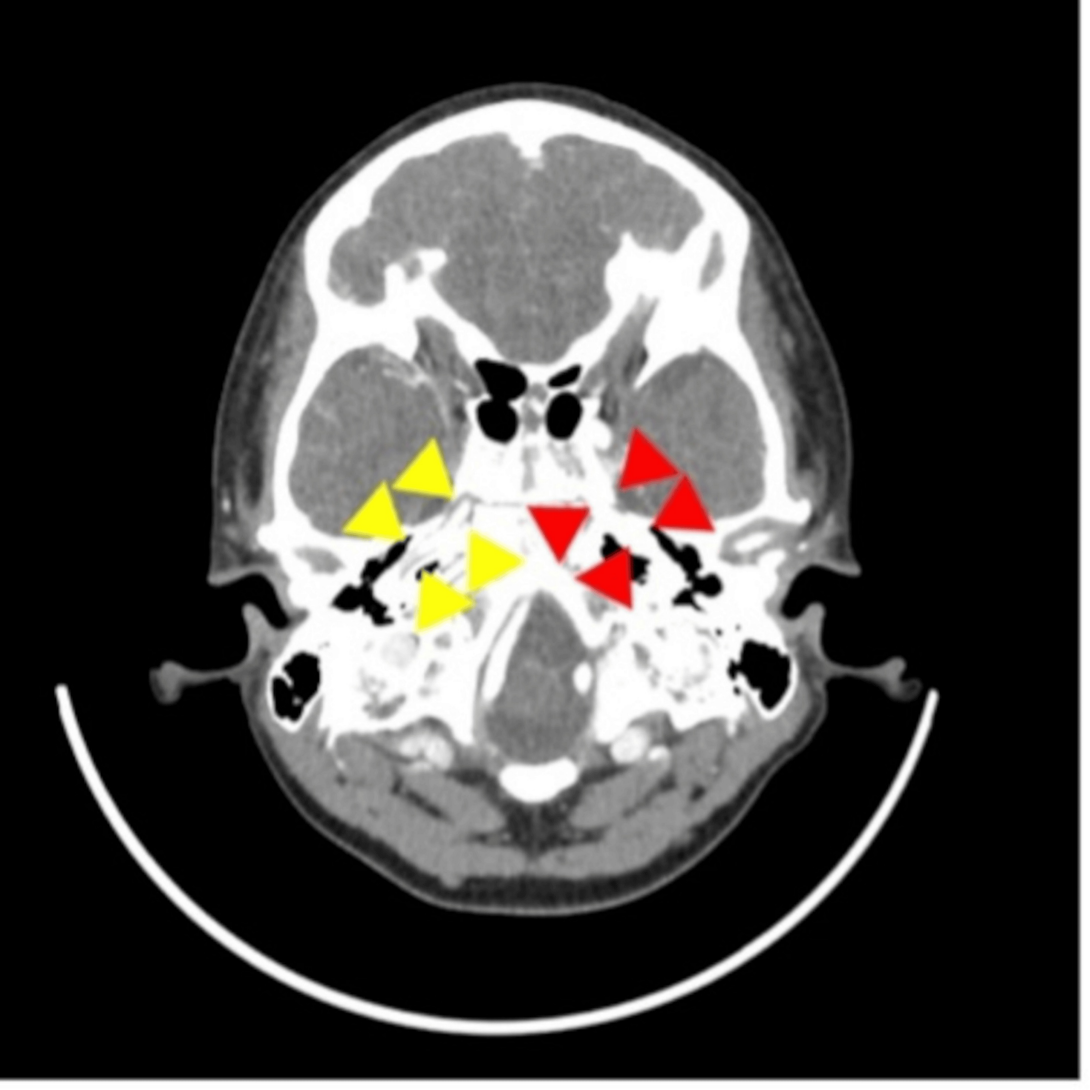
Contrast-enhanced computed tomography of the head on day 12 of admission. Contrast-enhanced computed tomography of the head on day 12 of admission shows circumferential thickening of the vessel wall of the right middle cerebral artery (yellow arrowheads). There is no contralateral vessel wall thickening (red arrowheads).

As a result, the patient was diagnosed with cerebral infarction. Low-molecular-weight dextran and edaravone were administered as acute treatments [5,6]. After confirmation of cerebral infarction, the results of general blood tests, including coagulation tests, were normal, and varicella zoster virus antibody titers and autoantibodies (antinuclear antibody, anti-cyclic citrullinated peptide antibody, and lymphocytotoxic antibody) were negative. The only abnormality observed was an elevated erythrocyte sedimentation rate (42 mm/h). On the evening of the second day of admission, the patient was able to kneel, shake hands, etc., and rapidly recovered to standing, holding a standing position, and walking. Edaravone and low-molecular-weight dextran were discontinued on days 7 and 9, respectively, after the rapid recovery of neurological findings and blood flow, as confirmed by MRA of the head on day 7 of admission. On day 12 of admission, contrast-enhanced CT and MRI scans of the head were conducted to investigate the cause of the cerebral infarction. The diagnosis of PCNSV was made based on circumferential wall thickening of the right middle cerebral artery (Figures 4-5).

Reversible cerebral vasoconstriction syndrome (RCVS) is a rare disease that is difficult to differentiate from idiopathic central vasculitis [2]. It is characterized by the acute onset of severe headache, referred to as thunderclap headache, which may be accompanied by neurological symptoms due to reversible vasoconstriction of the cerebral arteries. It commonly affects middle-aged women and can be induced by vasoactive and serotonergic drugs as well as the postpartum period. Furthermore, it may be accompanied by neurological symptoms such as convex subarachnoid hemorrhage, cerebral infarction, cerebral edema, and seizures several weeks after the onset of symptoms. Angiography shows bead-like changes with vasoconstriction and dilation in several areas of the cerebral arteries. In addition, severe headache often resolves in about one month, and vascular findings disappear in about three months. In this case, RCVS was ruled out due to the following reasons: there was only circumferential thickening of the right middle cerebral artery, no bead-like changes were observed, the thickening was limited to a single area, there were no multiple areas affected, and there was no prolonged headache.

Although immunosuppressive agents were considered for PCNSV treatment, the present case was nonprogressive and continued rehabilitation only as the neurological symptoms had already significantly improved at the time of diagnosis. After rehabilitation, it was confirmed that the patient had no problems with activities of daily living. Thus, he was discharged on day 18 of admission.

After discharge from the hospital, higher-order poststroke functional impairments such as irritability and attention deficit hyperactivity disorder-like behavior were noted. However, at four years following stroke onset, a repeat head MRI every six months revealed no evidence of recurrence, and neurological symptoms did not worsen.

## Discussion

An early school-age boy was brought to the hospital due to seizures and impaired consciousness after a fall. A simple CT scan revealed no abnormality, and his symptoms improved. However, on day 2 of admission, focal symptoms appeared and he was diagnosed with cerebral infarction. The cause of the stroke was determined to be PCNSV based on increased inflammatory response and circumferential wall thickening of the right middle cerebral artery as revealed by contrast-enhanced CT scan of the head. As the patient was diagnosed on day 12 of admission and had already shown marked improvement in neurological symptoms, no immunosuppressive therapy was added, and he was followed up without new neurological findings.

Cerebral infarction in children is a rare condition, occurring in 1 to 6 per 100,000 children, and the risk factors differ between children and adults [7]. The risk factors in children are attributed to various underlying conditions, such as moyamoya, cardiac, hematologic, and infectious diseases as well as vasculitis, and identification and treatment of the underlying condition are recommended. PCNSV is a type of vasculitis that is not associated with systemic vasculitis and is limited to the blood vessels of the central nervous system. The gold standard for the diagnosis of this disease is brain biopsy; however, a definitive diagnosis is difficult owing to its invasiveness and low sensitivity, with negative findings observed in approximately 50% of cases [8]. PCNSV can be broadly classified into angiography-positive and angiography-negative PCNSV, with the former typically involving the middle and large cerebral vessels with a stroke phenotype and the latter involving small vessels with an encephalitis phenotype. Angiography-positive PCNSV can be further divided into progressive and clinically nonprogressive subtypes [9], but these are often difficult to distinguish in the early stages of the disease. In advanced cases, treatment is recommended due to the poor prognosis, particularly immunosuppressive therapy with methylprednisolone or cyclophosphamide [10]. In the present case, clinical examination revealed a mildly elevated inflammatory response, but a contrast-enhanced CT scan revealed no evidence of systemic vascular inflammation. Furthermore, inflammatory findings were noted only in the right middle cerebral artery. Although treatment of cerebral infarction requires treatment of the underlying disease in parallel with conservative therapy, the patient exhibited rapid neurological recovery at the time of diagnosis, and MRA of the head revealed evidence of blood vessel reopening. Therefore, we established a treatment plan of careful follow-up without administration of immunosuppressive agents while continuing rehabilitation as the present case was considered to be clinically nonprogressive; the patient’s health continued to improve without further deterioration. Therefore, for patients with PCNSV with inflammation only in the medium and large vessels, such as our patient, who was suspected to have clinically nonprogressive PCNSV, follow-up with supportive care alone may be considered as a treatment option instead of immunosuppressive therapy, which has side effects. However, whether conservative treatment alone is sufficient in any given case needs to be evaluated as more cases accumulate in the future. It can also be inferred that the clinical course of the present case was similar to that of RCVS as headache was a symptom at onset and the patient had a transient, untreated course. PCNSV and RCVS share many similarities, and some patients diagnosed with RCVS may also have clinically nonprogressive PCNSV.

## Conclusions

The causes of pediatric stroke are diverse, and it is important to identify and appropriately treat the underlying disease. The clinical presentation of idiopathic central vasculitis is highly variable, and mild, clinically nonprogressive cases may not require immunosuppressive therapy. In addition, some cases of clinically nonprogressive PCNSV and RCVS may share similar clinical features.
